# Surveys to substantiate elimination of trachoma as a public health problem in the Republic of the Union of Myanmar

**DOI:** 10.1093/inthealth/ihaf038

**Published:** 2025-09-10

**Authors:** Mg Mg Myo Wynn, Ye Win, Ye Lin, San San Win, Thapa Badri, Jamsheed Mohamed, Lin Zaw, Anthony W Solomon, Michael Dejene, Caleb Mpyet, Yilikal Adamu, Aemero Abateneh, Mohammed Shafi, Cristina Jimenez, Ana Bakhtiari, Sarah Boyd, Anna Harte, Emma M Harding-Esch, Jeremiah M Ngondi

**Affiliations:** Ministry of Health, Nay Pyi Taw, Myanmar; Ministry of Health, Nay Pyi Taw, Myanmar; Ministry of Health, Nay Pyi Taw, Myanmar; World Health Organization, Yangon, Myanmar; World Health Organization, Yangon, Myanmar; World Health Organization, New Delhi, India; World Health Organization, New Delhi, India; Global Neglected Tropical Diseases Programme, World Health Organization, Geneva, Switzerland; Sightsavers, Addis Ababa, Ethiopia; Department of Ophthalmology, University of Jos, Jos, Nigeria; Sightsavers Nigeria Country Office, Kaduna, Nigeria; Addis Ababa University, Addis Ababa, Ethiopia; Jimma University, Jimma, Ethiopia; Adama Medical College, Adama, Ethiopia; Sightsavers, Haywards Heath, UK; International Trachoma Initiative, Task Force for Global Health, Decatur, Georgia, USA; International Trachoma Initiative, Task Force for Global Health, Decatur, Georgia, USA; London School of Hygiene and Tropical Medicine, London, UK; London School of Hygiene and Tropical Medicine, London, UK; RTI International, Washington DC, USA

**Keywords:** Myanmar, public health problem, trachoma survey, tropical data, validation of elimination

## Abstract

**Background:**

To determine whether trachoma elimination thresholds have been met in Myanmar, surveys were undertaken in 2019 to estimate the prevalence of trachomatous inflammation—follicular (TF) in children ages 1–9 y and the prevalence of trachomatous trichiasis (TT) unknown to the health system in adults ≥15 y of age.

**Methods:**

Surveys were conducted in 14 townships (evaluation units [EUs]) that were considered formerly endemic for trachoma. A two-stage cluster survey design was applied, whereby 30 villages and 30 households per village were sampled. Consenting eligible participants ≥1 y of age were examined for trachoma using the World Health Organization (WHO) simplified trachoma grading system.

**Results:**

A total of 12 594 households were surveyed, with a total of 5901 children ages 1–9 y and 31 097 people ≥15 y of age examined. In all 14 EUs, the prevalences of TF and TT unknown to the health system were below the elimination thresholds of <5% and <0.2%, respectively.

**Conclusions:**

Surveys showed that trachoma was no longer a public health problem in Myanmar and trachoma elimination thresholds had been attained. Partly on the basis of these data, in September 2020, Myanmar was validated by WHO as having eliminated trachoma as a public health problem.

## Introduction

Trachoma is the leading infectious cause of blindness. It is targeted for global elimination as a public health problem through the surgery, antibiotics, facial cleanliness and environmental improvement (SAFE) strategy.^1^

Trachoma control efforts in Myanmar predate the present-day SAFE strategy. The Myanmar trachoma control program was initiated in 1964 based on findings from a series of surveys undertaken in the 1960s and 1970s. The program covered 11 contiguous districts in the central ‘dry zone’ with a target population of 6.2 million people^2–4^ and was implemented in accordance with prevailing (1973) World Health Organization (WHO) guidance.^5^ The interventions consisted of surgical repair of trichiasis/entropion, topical antibiotic treatment with tetracycline eye ointment (TEO) for active trachoma and health education. Activities were carried out in three successive phases: the attack phase, in which TEO was given to the entire population in areas in which the prevalence of active trachoma was ≥30%; the consolidation phase, in which TEO courses were repeated whenever the prevalence of active trachoma was ≥15%; and the maintenance phase, in which only selective treatment for active trachoma was undertaken.^4^ A key intervention that was also continually implemented was school-based health education on facial cleanliness, which was integrated within the school health program and the teacher training curriculum. The program was primarily funded by the Ministry of Health, with additional funding from WHO, UNICEF, the United Nations Development Programme and a number of non-governmental organizations and implemented from 1966 onwards.^4^

At its inception, the Trachoma Control Project was a vertical program designed to be in operation for a short time, with eventual integration into basic health services. Following a decrease in the prevalence of trachoma accompanying implementation, the program evolved from control to surveillance and integrated its activities into basic health services as planned. In 1985, the program was renamed Trachoma Control and Prevention of Blindness (TC&PBL), with an expanded remit to prevent and manage other causes of blindness in addition to trachoma.^2^ By the early 1990s, the program employed 400 staff dedicated to trachoma control and covered approximately 15 million people in 82 townships across 14 districts.^4^ The Ministry of Health then began shifting from a vertical program to an integrated program (in which trachoma screening and treatment were included in routine primary eye care activities).^4^ Trachoma control in Myanmar entered the surveillance phase in the early 2000s. After >5 decades of trachoma control activities in Myanmar, it is estimated that >6.5 million people received TEO mass drug administration and >150 000 people who had trichiasis received surgery.^6^

To determine whether elimination prevalence thresholds had been met, new baseline surveys were needed.^7^ These surveys were undertaken in 2019, with the objective of estimating the prevalence of trachomatous inflammation—follicular (TF) in children ages 1–9 y, the prevalence of trachomatous trichiasis (TT) unknown to the health system in adults ≥15 y of age and the prevalence at the household level of access to water, sanitation and hygiene (WASH).

## Methods

### Study sites

Historically, trachoma in Myanmar was considered a disease of the dry, hot, central Irrawaddy River plain, stretching from districts near Mandalay south to Magway. Trachoma was known to be non-endemic in the hilly, mountainous and coastal areas.^8^ By 2019, the trachoma control catchment area comprised 14 townships across three regions (Magway, Mandalay and Sagaing).^2–4^ The catchment areas were determined based on results from the 2018 national Rapid Assessment of Avoidable Blindness (RAAB) survey across 18 states/regions^9^ that showed blindness due to trachoma was most common in Magway and Sagaing. The RAAB findings were triangulated with results of a survey on reported trichiasis cases completed by each regional ophthalmologist.^10^ Each of the 14 townships was defined as a distinct evaluation unit (EU) for the new baseline population-based prevalence surveys; urban areas were not considered to be trachoma endemic and were therefore excluded from the sampling frame in each EU.^10^

### Sample size estimation

Since trachoma was not suspected to be a public health problem in any surveyed EU, sample size estimation was based on the elimination threshold for TF in those aged 1–9 y. To estimate the EU-level prevalence of TF among children ages 1–9 y, the sample size was calculated aiming to have 95% confidence of estimating an expected prevalence of 4% with absolute precision of ±2%, using a design effect of 2.63 and a non-response inflator of 1.2.^7^ Based on these parameters, a sample of at least 1164 children ages 1–9 y was required to be enumerated per EU.

Each survey team was expected to be able to cover 30 households per day. Therefore, to meet the required sample size, 30 households in each of 53 clusters (defined as villages) were needed. However, 30 clusters were selected following WHO recommendations that this number of villages provides adequate precision around the TF prevalence estimate; this also allowed for sufficient confidence in the precision of the TT prevalence estimates.^11^

### Sample selection

Two-stage cluster sampling was used. Villages were used as first-stage clusters. A list of villages in the rural wards of each EU was obtained from relevant local government offices and 30 villages were systematically selected with probability proportional to village population size. In the second stage, the compact segment sampling technique was used to select 30 households in each selected village. All people ≥1 y of age residing in selected households were invited to participate.

### Training of survey teams

The survey teams comprised graders and recorders who were trained and certified using methods developed by the Global Trachoma Mapping Project^12,13^ and Tropical Data (www.tropicaldata.org).^14–16^ Trachoma grader-trainees were trained on the WHO simplified grading system^17^; photo-based intergrader agreement (IGA) tests were done in Shwebo, Myanmar. Because there were insufficient numbers of TF cases in Myanmar to complete grader training in the field in-country, seven grader-trainees who had passed the photo-based IGA tests underwent further field-based training in a trachoma-endemic area of Hawassa, Ethiopia, in August 2019.^18^ Field-based IGA tests were conducted using sets of 50 children in Ethiopia. All seven grader-trainees achieved κ scores for TF of at least 0.7 (range 0.74–0.95) compared with the certified Tropical Data principal grader and were thus eligible to participate in the surveys in Myanmar. Survey data recorders were trained in Myanmar on electronic capture of survey data using the Tropical Data system (www.tropicaldata.org) and reliability tests were undertaken and passed. Having completed the grader and recorder training, trainees who qualified were paired into six teams of graders and recorders for further team training in Myanmar. The senior-most grader was appointed as supervisor.

### Household interviews

Household-level data on WASH were collected by trained interviewers using a standard questionnaire.^12,15^ Heads of households were interviewed on types of water sources, distance to water sources and type of sanitation facilities used by the household; when the family reported using a latrine, its existence and type were verified through observation.

### Trachoma examination

The eyelids and tarsal conjunctivae were examined using a ×2.5 magnifying loupe and torchlight for signs of active trachoma and its complications. TT was defined (using the 1987 definition^17^) as the presence of at least one eyelash from either the upper or lower eyelid touching the eyeball or evidence of recent epilation of in-turned eyelashes. For eyes with TT, the tarsal conjunctiva was examined for trachomatous scarring (TS) and the patient was asked questions about whether he/she had previously been offered trichiasis surgery or epilation by a healthcare worker for that eye. Follicle size guides were used to assist with standardised diagnosis of TF.^19^ People identified as having active trachoma or conjunctivitis with a presumed bacterial cause were provided with 1% TEO, regardless of age. Individuals with TT or other eye conditions were referred to the appropriate secondary eye care health facilities for further evaluation and management. The survey teams returned to households at the end of each day to examine household members from the target age group (1-9-year-olds are the target age group for baseline surveys; TF in this age group is what the surveys are powered for) who were absent at the time of the initial examination.

### Data management and analysis

Data were collected electronically using the Tropical Data system and analyzed using the methods of the Global Trachoma Mapping Project and Tropical Data.^12,15,16^ Questions about previous offers of surgery or epilation enabled estimation of the proportion of TT patients who were unknown to the health system; estimates of the prevalence of TT reported here include all trichiasis, ignoring the presence or absence of TS. Age- and gender-specific weights were calculated based on the 2014 population census^20^ and applied to survey data. Age- and gender-adjusted point prevalence estimates and confidence intervals were generated for TF in children ages 1–9 y and TT unknown to the health system in adults ≥15 y of age.

### Ethical issues

The study was approved by the Research and Ethics Board of Health, Ministry of Health, Myanmar. Ethical approval for Tropical Data to provide support for these surveys was granted by the London School of Hygiene & Tropical Medicine (16105). Permission for households to be enrolled in the surveys was sought from the heads of each selected household. Informed verbal consent for examination was sought from each survey participant ≥18 y of age or, in the case of people <18 y of age, their parent or guardian. Additionally, children ages 6–17 y were asked to assent to take part. Consent for examination was recorded in the Tropical Data app used for data collection.

## Results

### Sample population

Fieldwork took place from September to December 2019. Tables 1 and 2 summarize the sample population by district. A total of 12 594 households were surveyed from 420 villages in 14 townships (EUs). Survey participants included 5901 (98% of enumerated) children aged 1–9 y and 31 097 (81% of enumerated) people ≥15 y of age.

**Table 1.** Prevalence of trachomatous inflammation—follicular (TF) and trachomatous trichiasis (TT) by EU, Myanmar, 2019

			Children ages 1–9 y			Adults ≥15 y		
Region	District	Township	Number enumerated	Number examined	Prevalence of TF, % (95% CI)	Number enumerated	Number examined	Prevalence of TT^a^ unknown to the health system, % (95% CI)
Sagaing	Sagaing	Sagaing	442	425	0.0	2875	2206	0.13 (0.04 to 0.24)
Sagaing	Shwebo	Shwebo	411	407	0.1 (0 to 0.3)	2879	2494	0.15 (0.02 to 0.32)
Sagaing	Monywa	Monywa	420	417	0.0	2902	2374	0.04 (0.0 to 0.09)
Magway	Magway	Magway	431	420	0.0	2471	2141	0.06 (0.0 to 0.19)
Magway	Magway	Chauk	476	469	0.0	2605	2233	0.03 (0.0 to 0.07)
Magway	Magway	Taungdwingyi	388	385	0.0	2648	2253	0.04 (0.0 to 0.11)
Magway	Minbu	Minbu	441	433	0.0	2447	2144	0.08 (0.01 to 0.18)
Magway	Pakokku	Pakokku	383	381	0.0	2747	2340	0.06 (0.01 to 0.13)
Magway	Pakokku	Myaing	466	464	0.0	2669	2225	0.12 (0.05 to 0.21)
Magway	Gangaw	Gangaw	417	406	0.0	2801	2430	0.00
Mandalay	Kyaukse	Sintgaing	481	477	0.0	2725	2075	0.09 (0.0 to 0.24)
Mandalay	Myingyan	Myingyan	400	387	0.0	2970	2256	0.14 (0.03 to 0.26)
Mandalay	Myingyan	Kyaukpadaung	394	375	0.0	2798	1937	0.07 (0.02 to 0.12)
Mandalay	Meiktila	Meiktila	479	455	0.1 (0 to 0.2)	2789	1989	0.04 (0.0 to 0.11)

CI: confidence interval.

a TT was defined as the presence of at least one eyelash (from the upper or lower eyelid) touching the eyeball or evidence of recent epilation of in-turned eyelashes.

**Table 2.** Proportion of households with access to water and sanitation, by EU, trachoma surveys, Myanmar, 2019

				Proportion of households (%)		
Region	District	Township	Households sampled, n	Using improved drinking water source^a^	With drinking water source in yard or within 1 km	With improved sanitation facility^a^
Sagaing	Sagaing	Sagaing	900	66	100	84
Sagaing	Shwebo	Shwebo	899	74	100	86
Sagaing	Monywa	Monywa	900	95	98	82
Magway	Magway	Magway	900	58	93	92
Magway	Magway	Chauk	900	82	99	79
Magway	Magway	Taungdwingyi	900	99	100	94
Magway	Minbu	Minbu	900	91	99	91
Magway	Pakokku	Pakokku	899	96	100	82
Magway	Pakokku	Myaing	900	95	100	91
Magway	Gangaw	Gangaw	899	86	100	94
Mandalay	Kyaukse	Sintgaing	900	73	100	84
Mandalay	Myingyan	Myingyan	900	89	99	73
Mandalay	Myingyan	Kyaukpadaung	899	75	90	71
Mandalay	Meiktila	Meiktila	898	80	94	50

a Definition of improved water sources and improved sanitation facilities were based on the WHO/UNICEF Joint Monitoring Program, available from: https://washdata.org/monitoring/methods/facility-types.

### Prevalence of TF and TT

**Figure 1.**
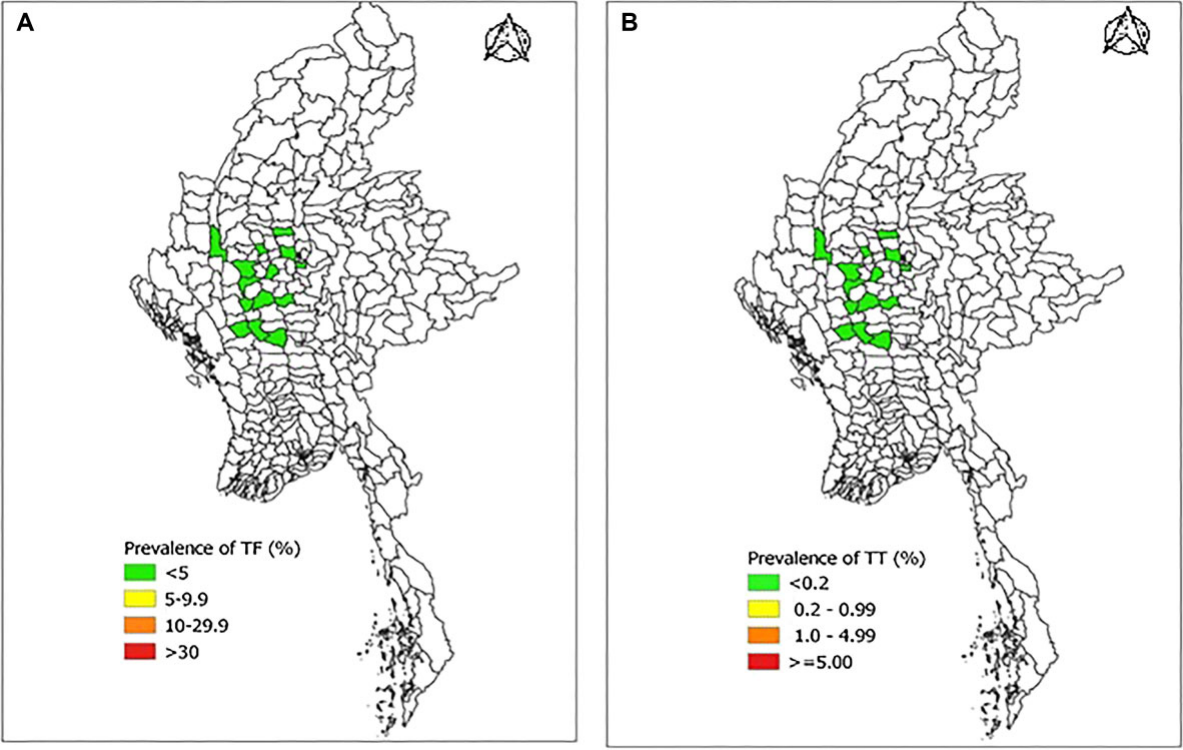
Prevalence of (a) trachomatous inflammation—follicular (TF) in 1- to 9-year-olds and (b) trachomatous trichiasis (TT) in adults ≥15 y old, by EU, Myanmar, 2019. The boundaries and names shown and the designations used on this map do not imply the expression of any opinion whatsoever on the part of the authors or the institutions with which they are affiliated concerning the legal status of any country, territory, city or area or of its authorities or concerning the delimitation of its frontiers or boundaries

Table 1 and Figure 1 show the prevalence of TF and TT in each EU. In each EU, the prevalence of TF was <5% and the prevalence of TT unknown to the health system was <0.2%.

### Household access to WASH

The proportions of household-level access to WASH (ranges by EU) were improved drinking water source, 58–99%; drinking water source in the yard or within 1 km, 90–100%; and improved sanitation facility, 50–94% (Table 2).

## Discussion

Surveys showed that trachoma was no longer a public health problem in Myanmar and trachoma elimination thresholds had been attained.^21^ In addition, Myanmar's TC&PBL program has established a robust system for identification of TT cases and provision of TT surgery services through primary (PECs) and secondary eye care centres (SECs)^6^ located in formerly trachoma-endemic areas. In regions/states outside the formerly trachoma-endemic areas, examination for trachoma was integrated into routine primary eye care with referral of TT cases to SECs and tertiary eye care centres for TT surgery. The PECs and SECs focus on providing comprehensive eye care, with examinations for cataract, glaucoma, refractive errors, active trachoma, trichiasis and other ocular disease pathologies. Nationally, data on the number of people examined, treated and operated on during PEC activities (village, school and outpatient eye health examinations) are collated monthly and reported to the program through the SECs. The national eye health program has outlined the objectives and resources that will be needed to maintain elimination of trachoma as a public health problem, including ongoing identification of TT cases and provision of TT surgery through PECs and SECs. This latter point demonstrates the existence of a system able to identify and manage incident TT, as required by WHO. Based on this evidence, the results of the population-based surveys and submission of a dossier to WHO,^21^ Myanmar was validated for elimination of trachoma as a public health problem in September 2020.^8^

This series of surveys used WHO-recommended methods for population-based surveys of trachoma^7^ and was implemented using Tropical Data's systems and standards.^16^ Since trachoma elimination is a reversible state,^21^ the TC&PBL program has established an ongoing national surveillance system through the aforementioned PEC and SEC services.^6,22^ Surveillance for trachoma is underpinned by routine training of all eye care workers on trachoma examination using a standard manual developed by the TC&PBL,^23^ following the simplified trachoma grading system as recommended by WHO.^24^ Training to diagnose TF and TT is now being enhanced with photographs, given that cases of TF and TT are now rare in the country. Trachoma examination is part of a well-established routine eye care examination that monitors and reports on a range of eye diseases as previously described, and therefore costs are integrated into the entire eye health system. Data on the number of people examined, treated and operated on for trachoma during primary and secondary eye care services are collated monthly and reported to the programme.^22^ Since 2010, the proportion of children <10 y old with active trachoma in each region/state has been routinely reported in the annual public health statistics and has consistently remained below the elimination thresholds^25^ for the entirety of Myanmar. The well-established and routine monitoring of trachoma means post-elimination surveys will not be required.

In addition, Myanmar has continued to sustain trachoma prevention activities through extended, multilevel interventions involving health education and improved access to WASH.^26^ Health education on facial cleanliness is still undertaken in schools and in the community. School-based health education uses the slogan ‘clean hands, clean feet and clean faces for good health’.^8^ This behaviour change communication is integrated within the school health program and is also part of the school curriculum. The WASH program is implemented by several government ministries and departments, with support from national and international non-governmental organizations.^27^ The survey findings on access to WASH in the 14 previously trachoma-endemic townships (Table 2) provides evidence of efforts to maximize access to WASH. Compared with the most recent Demographic and Health Survey (2014–2015), across all 14 EUs, households’ access to an improved drinking water source was consistent with the national average (80%) while households with an improved sanitation facility was above the national average (48%).^28^ Multicountry analyses suggest that communities in which >80% of households have access to improved sanitation enjoy herd protection against active trachoma.^29^

Regionally, Myanmar is unlikely to be at risk of cross-border reintroduction of active trachoma from neighbouring countries. India,^30^ China^31^ and Lao People's Democratic Republic^32^ have each been validated as having eliminated trachoma, while Bangladesh and Thailand are ‘thought to not require interventions’.^33^ India has now been validated as having eliminated trachoma as a public health problem.

These surveys followed standard WHO trachoma survey methodologies; however, there are several limitations. First, the sample sizes for TF were less than half of what is recommended per EU due to the number of clusters being reduced from the initial estimate of 53 to the maximum recommended number of clusters of 30. Nonetheless, previous analyses published by WHO demonstrate that including >30 clusters per EU results in only marginal increases in precision.^7,11^ Thus we are confident that the results reflect reliable population-level estimates based on the WHO standards.^7^ Second, while the proportion of children aged 1–9 y who were examined was quite high (98% overall), the proportion of adults ≥15 y of age was lower, ranging from 69% to 88% by EU. Most of the persons ≥15 y of age not examined were men, who were absent from the households mainly for work duties. While this is a potential source of bias, we do not expect this affected our conclusions of attainment of TT prevalence <0.2% in all EUs since men are less likely to have TT compared with women.^34^

## Conclusions

The surveys done in 2019 and described in this article provide a vital evidence base for substantiating elimination of trachoma as a public health problem in Myanmar. In addition, Myanmar has established a system for ongoing identification of TT cases and provision of TT surgery. Most importantly, Myanmar has sustained interventions for prevention of trachoma and has established a surveillance system for active trachoma and trichiasis.

## Data Availability

The data are available upon request from the Myanmar Ministry of Health.

## Notes

**© 2024 World Health Organization. Licensee Taylor & Francis.**

This is an open access article distributed under the Creative Commons Attribution IGO License, which permits unrestricted use, distribution, and reproduction in any medium, provided the original work is properly cited: https://creativecommons.org/licenses/by/3.0/igo/. In any use of this article, there should be no suggestion that WHO endorses any specific organization, products or services. The use of the WHO logo is not permitted. This notice should be preserved along with the article's original URL.
